# Evaluating the integration of chronic disease prevention and management services into primary health care

**DOI:** 10.1186/1472-6963-13-132

**Published:** 2013-04-08

**Authors:** Martin Fortin, Maud-Christine Chouinard, Tarek Bouhali, Marie-France Dubois, Cynthia Gagnon, Martin Bélanger

**Affiliations:** 1 Département de médecine de famille, Université de Sherbrooke, Québec, Canada; 2 Centre de santé et de services sociaux de Chicoutimi, 305, St-Vallier, Québec, G7H 5H6, Canada; 3 Département des sciences de la santé, Université du Québec à Chicoutimi, Québec, Canada; 4 Faculté de médecine et des sciences de la santé, Université de Sherbrooke, Québec, Canada; 5 Centre de santé et de services sociaux de Jonquière, Québec, Canada; 6 Agence de la santé et des services sociaux du Saguenay-Lac-Saint-Jean, Québec, Canada

## Abstract

**Background:**

The increasing number of patients with chronic diseases represents a challenge for health care systems. The Chronic Care Model suggests a multi-component remodelling of chronic disease services to improve patient outcomes. To meet the complex and ongoing needs of patients, chronic disease prevention and management (CDPM) has been advocated as a key feature of primary care producing better outcomes, greater effectiveness and improved access to services compared to other sectors. The objective of this study is to evaluate the adaptation and implementation of an intervention involving the integration of chronic disease prevention and management (CDPM) services into primary health care.

**Methods/Design:**

The implementation of the intervention will be evaluated using descriptive qualitative methods to collect data from various stakeholders (decision-makers, primary care professionals, CDPM professionals and patients) before, during and after the implementation. The evaluation of the effects will be based on a combination of experimental designs: a randomized trial using a delayed intervention arm (n = 326), a before-and-after design with repeated measures (n = 163), and a quasi-experimental design using a comparative cohort (n = 326). This evaluation will utilize self-report questionnaires measuring self-efficacy, empowerment, comorbidity, health behaviour, functional health status, quality of life, psychological well-being, patient characteristics and co-interventions. The study will take place in eight primary care practices of the Saguenay region of Quebec (Canada). To be included, patients will have to be referred by their primary care provider and present at least one of the following conditions (or their risk factors): diabetes, cardiovascular diseases, chronic obstructive pulmonary disease, asthma. Patients presenting serious cognitive problems will be excluded.

**Discussion:**

In the short-term, improved patient self-efficacy and empowerment are expected. In the mid-term, we expect to observe an improvement in health behaviour, functional health status, quality of life and psychological well-being. At the organizational level, the project should lead to coordinated service delivery, improved patient follow-up mechanisms and enhanced interprofessional collaboration. Integration of CDPM services at the point of care in primary care practices is a promising innovation in care delivery that needs to be thoroughly evaluated.

**Trial registration:**

ClinicalTrials.gov Identifier: NCT01319656

## Background

The last decades have seen dramatic shifts in the pattern of diseases from infectious diseases to the current leading causes of mortality dominated by chronic diseases (CD) accounting for 59% of annual deaths and 46% of the global burden of disease [1]. Research suggests that complex chronic conditions such as diabetes, cardiovascular and respiratory diseases will impose an even larger burden in the future [1,2]. This represents a challenge for the health care system. While traditional health care funding and management are mainly designed to address acute health conditions, the bulk of health funds are allocated to patients with CD who are the heaviest users. In Canada, it is estimated that 42% of total direct medical care expenditures ($39 billion) are used each year to treat people with CD [3]. Due to lack of coordination of care and difficulties accessing services, patients with CD are disadvantaged in the current health care system and are, together with physicians, decision-makers and the general population, looking for fundamental changes. Several solutions have been proposed at the organizational and patient level.

From an organizational point of view, it has been demonstrated that a strong primary health care system is associated with better health indicators and more sustainable costs [4-9]. A recent report of the Canadian health care authorities has put forward the added-value of a strong primary health care system recognizing the role of primary care as a pivotal organization ensuring proper use of professional skills in the management of chronic diseases [10]. However, patients with CD represent a greater challenge to primary health care as they are associated with high health care costs and poor compliance to treatment and recommendations [11-13]. In the presence of CD, primary health care providers face difficulties in applying guidelines [14,15] and in maintaining care continuity [16]. To prevent potential health care system gaps in quality, efficiency, and effectiveness, the Chronic Care Model (CCM) has been suggested as a promising solution. Hence, the integration and application of the CCM into primary care organizations should be supported [17,18]. Although various stakeholders do agree on the relevance of the CCM approach, translating its conceptual clusters into operational strategies is a complex undertaking. Consequently, decision-makers and physicians have to consider reliable evidence-based evaluations to improve quality of care and to manage allocation of resources as efficiently as possible [19].

Lifestyle-related risk factors of CD, such as obesity, physical inactivity and diet-related behaviours, have been linked to increased risks of morbidity and mortality [20]. This reflects the 40% of CD that can be prevented [21]. To meet the complex needs of patients with chronic conditions seen in primary care, these settings may benefit from being able to offer a range of chronic disease prevention and management (CDPM) services under one roof. Such integrated services have been identified as a potential key to fulfilling standards of care for patients with CD. To prevent primary health care clinics from operating in isolation, CDPM services have to be provided by an interdisciplinary team in coordination with primary care. Interdisciplinary teamwork has been associated with a higher use of preventive strategies and a lower burden for caregivers [22]. Primary care physicians are in the best position to coordinate these strategies as they see patients more frequently and at earlier stages of the disease than specialists. In the search for strategies that might help reduce the burden of CD and related high risk lifestyles, resource utilization and management schemes deserve to be evaluated. This makes evaluation an integral part of the implementation process.

In the Saguenay-Lac-Saint-Jean region of Québec (Canada), health care professionals have been mobilized to deal with the challenges of CD management through the introduction of integrated CDPM services. Since 2001, a strong network of CDPM services has been deployed in the six health care centers across the region. The approach groups together various CD based on common CDPM services, with a range of standardized services rooted in evidence-based medicine and integrated at the local level into each of the six regional territories [23,24]. The program addresses a number of CD, including cardiovascular disease (CVD), heart failure, chronic obstructive pulmonary disease (COPD), asthma and diabetes, and aims to: (1) reduce and correct modifiable risk factors; (2) strengthen individual self-efficacy; (3) optimize functional autonomy, biopsychosocial balance and health; and (4) support self-management [23]. The program offers a range of activities (educational, counselling, follow-up) delivered by various professionals on aspects such as compliance with medication/vaccination, nutrition, physical activity, smoking cessation, stress management and psychosocial support [23]. The variety of services for a given patient are scheduled over a six-month period and may include individual or group meetings [25]. The program intervention principles are based on complementary theoretical models: McGill’s nursing conceptual model [26,27], the PRECEDE model [28,29], and the Prochaska & DiClemente stages of change model [30,31]. However, collaboration between this network and the primary care providers has so far not been optimal for several reasons and consequently the services have not reached a large part of the population with CD. Services were offered to people mainly after an acute episode of care or hospitalization.

The Cochrane Collaboration published a review of the effectiveness of care and services shared between primary care services and specialized care for CD management; however it was not possible to draw conclusions regarding the effectiveness of integration interventions in this field at the time [32]. Theoretically, this kind of shared care model presents an opportunity to provide patients with the benefits of specialist intervention combined with continuity of care and management of comorbidity more easily offered through primary care whose providers maintain responsibility for all aspects of patient health care beyond a single specific CD. In this regard, a recent Canadian experience with interventions involving close collaboration between primary care settings and CD management services stressed the desirability of this integration [33,34]. The implementation in Alberta of an integrated services network within primary care settings, based on close collaboration with specialized services, demonstrated a positive impact on patients, professionals and the organization of services. However, the total effects of this intervention are still being investigated.

CDPM has been shown to be effective in the context of CVD [35], COPD [36], asthma [37] and diabetes [38]. Usually offered in specialized settings, such interventions have had different outcomes such as a decrease in the use of health services, and improved functional status and quality of life, most often in the short term. Overall, these studies were conducted outside primary care practices and were limited in time. Intervention models and theoretical foundations underlying these programs are varied, but most often were founded on a patient education intervention conducted by different health professionals specialized in CD and focused on supporting the modification of risk factors and self-management.

To date, the appropriateness of using multidisciplinary specialized professionals in the context of primary care practices has been little studied. The few studies found were conducted along the same lines as that of specialized CD interventions, that is, in the context of a single disease, such as COPD and asthma [39], metabolic syndrome [40], kidney failure [41], diabetes [42], or among people with risk factors [43]. Again, interventions were based on different approaches, mainly on self-management support and patient education. The outcomes used vary greatly from one study to another and include the use of services, quality of life, various physiological indicators and risk factor modification.

Among ongoing studies, Perula et al. published a study protocol of an open, two-arm parallel, non-pharmacological approach based on the promotion of a healthy diet and physical activity to control cholesterol levels among patients with hyperlipidemia in the context of primary care [44]. The intervention is based either on motivational interviewing or on the usual brief advice. The study will assess the degree of dietary and physical activity improvement, weight loss in overweight patients, adherence to treatment guidelines as well as lipid levels and cardiovascular risk.

**Figure 1:**
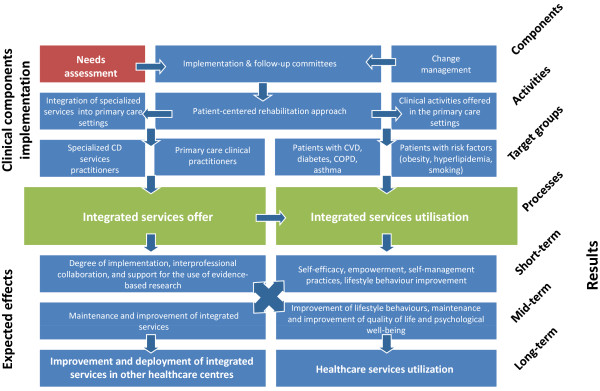
Intervention logic model.

This study aims to introduce a pragmatic innovation by adapting and integrating CDPM services into primary care settings and to propose an innovative combination of strategies to evaluate the effects and implementation of this intervention in eight primary care practices in the Saguenay region. The main components of this intervention are illustrated in the intervention logic model presented in Figure 1. The evaluation-specific objectives are to: (1) assess the implementation of a clinical intervention of CDPM services integrated into the existing structure of the primary care network in order to describe the implementation context, the implementation process and the satisfaction of those involved, including the perspective of patients; (2) measure the effect and the effectiveness of interventions among primary care patients. We hypothesize that patients receiving the intervention will report better empowerment and self-efficacy and will demonstrate reduced health risk behaviours.

## Methods/Design

### Settings

The intervention will be implemented in the Saguenay region of Quebec (Canada) where primary care physicians work in two types of organizations principally: Group practices, involving a group of primary care physicians working in a team with no access to professionals from other disciplines; Family Medicine Groups, new organizations in which primary care physicians work with nurses at various degrees of collaboration and share the same patients and medical files. No other disciplines are involved in the practice. Both organizations provide care for a population including a majority of patients with chronic diseases and risk factors.

### Intervention

The clinical components of the intervention consist of many steps described in the logic model developed for this project (Figure 1). According to the needs expressed by primary care professionals, a number of patient-centered activities were developed. CDPM services were adapted to services and resources already in place with a focus on self-management support, patient-centered care, motivational interviewing, interprofessional collaboration and integration of services. Such a multi-dimensional learning package is designed to improve both process and outcome measures and increase intervention impact. This model has proven to be effective in the development of behavioural interventions in patients with CD [45]. Further detail on the intervention is provided in Table 1.

**Table 1:** Characteristics of the interventions

**CDPM practitioner training**	Theoretical training on:
	− Motivational interviewing
	− Function of the respiratory system
	− Function of the cardiovascular system
	− Diabetes
	− Risk factors
	− Existing CDPM services
	Practical training:
	− Three-week mentoring in specialized CDPM services facilities.
**Preliminary clinical evaluation**	The clinical evaluation of participants includes:
	− Anthropometric characteristics
	− Medical history
	− Medication
	− Functions (respiratory, cardiovascular, endocrine, gastro-intestinal)
	− Lifestyle habits and risk factors
	− Patient preoccupations and objectives
	− Previous interventions (nutrition, physical activity, respiratory, smoking cessation)
	− Recent changes (weight, alcohol consumption)
**Disciplines involved in the intervention**	The interventions, based on a referral from a family physician or nurse, are provided by professionals in the following disciplines:
	− Clinical coordination
	− Nursing
	− Physical activity therapy
	− Nutrition
	− Respiratory therapy
	− Smoking cessation therapy
**Implemented interventions**	The interventions implemented are:
	− Self-management support
	− Education on diseases (diabetes, COPD, asthma, cardiovascular)
	− Education on risk factors (pre-diabetes, high blood pressure, dyslipidemias, obesity, physical inactivity, smoking)
	− Counseling on medication
	− Motivational interviewing
	− Education about nutrition
	− Education about physical activity
	− Counseling on smoking cessation
**Tools and support material**	Each intervention is supported by print and other material to ensure that patient engagement is maintained even between the interventions. These include documents on:
	− Chronic disease management
	− Asthma, COPD
	− Diabetes
	− Cardiovascular
	− Metabolic syndrome
	− Hypo/hypertension
	− Tools for smoking cessation
	− Stress management
	− Blood pressure monitoring journal
	− Personal objectives journal
	− Physical activity journal
**Communication & coordination**	The CDPM practitioners in our study work within primary care settings which enhances communication with primary care physicians, nurses and staff. The clinical coordinator ensures optimal communication and transition of care between the project team, the primary care professionals and specialized services. Special attention is given to the distinction of tasks fulfilled by project CDPM practitioners and tasks fulfilled by primary care nurses.
**Integration**	Prior to the implementation of interventions, a pre-implementation evaluation is conducted to identify the needs for CDPM services and the contextual factors of the participating PC clinics in the follow-up of CD patients. The pre-implementation evaluation of the project promotes the sharing of a common positive vision of an intervention that focuses on prevention, earlier support for patients in the course of their disease, interprofessional collaboration, services integration, motivational interviewing and self-management support.
**Participating primary care professionals**	Participating primary care professional include:
	− Family physicians (63)
	− Nurses (5)
	Participating primary care settings:
	− Four (4) clinics
	− Four (4) family medicine groups
**Participating specialists**	Participating specialists include:
	− Cardiologists
	− Internal medicine specialists
	− Endocrinologists
	− Pneumologists

The intervention will be implemented using support mechanisms and ongoing evaluation within the participating clinics to ensure a harmonious integration. Interventions at the patient level will: (a) be patient-centered and educational in nature; (b) last less than three months and involve at least three encounters with CDPM practitioners; (c) include individual interviews or patient focus groups involving close relatives. The intervention will consolidate the central role of primary health care professionals and their organizations, while maintaining the natural proximity between the patient and his or her family physician. In this regard, interventions will: (a) be carried out upon referral from the primary care team; (b) allow an exchange with the primary care team and will be recorded in primary care medical records; (c) return the responsibility of long-term follow-up to the primary care team.

### Evaluation

Consistent with the logic model (Figure 1), the evaluative approach will focus both on the implementation of the intervention and the measurement of its effects by using a mixed methods design [46] to evaluate health interventions [47]. The implementation evaluation will involve descriptive qualitative methods, while the evaluation of the effects will be based on quantitative methodology. This combination of approaches used concomitantly aims to deepen our understanding and to corroborate the evaluation results.

#### Implementation evaluation (objective 1)

Implementation evaluation consists in investigating the relationship between an intervention and its context during implementation [48]. It will be based on two approaches: a realistic evaluation, aiming to explain how various contexts influence observed effects [49], and a participatory evaluation aiming to determine the elements that could potentially inform and help improve future interventions and decision-making [50]. A realistic evaluation recognizes that any effect (E) of an intervention arises from the interaction between the intervention (I) and the context of its deployment (C). It aims to highlight the underlying mechanisms as well as the way they operate under certain conditions. Information about these three elements (E, I and C) will be collected during all three stages of the implementation. Moreover, realistic evaluation recognizes that results can be apparent not only at patient-level but also at the level of the professionals and organizations involved [51].

The participatory evaluation approach will help to adapt both the data collection tools and the data analysis strategy in order to bring to light elements that can guide future intervention implementations.

Table 2 describes the implementation evaluation focus (context, intervention or effects) that consists of three phases (pre-, per- and post-intervention) and will be conducted among the five categories of partners involved in the project (decision-makers, n = 15; primary care physicians, n = 63; clinics, n = 8; specialists, n = 12; CDPM professionals, n = 20; patients and their families, n = 326). Patients (5 per clinic, total = 40) and their families (spouse or main family caregiver) will be invited to take part in focus groups.

**Table 2:** Implementation evaluation

**Evaluated dimensions**	**Decision-makers**	**Primary care prof.**	**Specia-lists**	**CDPM prof.**	**Patients/family**	**Patient records**	**Project documentation**
**Pre-intervention stage**							
Description of settings (contextual factors) (C)	FG	FG	II	FG			
Needs analysis (C)	FG	FG	II	FG			
**Intervention stage**							
Identification of problems and difficulties (C)							Document analysis
Care and services for patients (I) (Intervention fidelity)						Checklist entry	
**Post-intervention stage**							
Extent of implementation/Services offered (I)				II		Data entry	
Opinion on the implementation process (C)	FG	FG		FG			
Description of effects on professionals/organizations (E)	FG	FG		FG			
Care and services for patients (I)				FG	FG		
Identification of barriers and facilitating factors (C)	FG	FG		II			
Satisfaction with intervention (E)	FG	FG		II	FG		

FG: Focus group; II: Individual interview; C: Context; I: Intervention and action mechanisms; E: Effects of the intervention at the patient, practitioner and organizational levels.

To ensure fidelity of the intervention and to prevent clustering, the intervention was designed by the research team. Nevertheless, in this regard the research team consulted with primary health care providers in each practice, specialists, and decision-makers to ensure that the intervention is adapted to the needs expressed. Moreover, interventions will be done by CDPM professionals, recruited and trained by the research team about the general principles of self-management support, patient-centered care, motivational interviewing, interprofessional collaboration and integration of services. The rationale of such a process is to ensure standardization of practices within and across the eight clinics in order to prevent study interventions from evolving differently, as results will be unusable if participants experience different interventions. However, despite the use of such patterns, heterogeneity that may be observed and documented between clinics in terms of outcomes produced, termed “center effects” is part of the pragmatic design and will reflect the clinical reality of primary health care. It has been suggested that interventions may be affected by center-specific issues and characteristics such as degree of sub-specialization within practices and provider background and experience, [52] however, we believe that delivering interventions by practitioners who evolve through the same training process will reduce clustering effects so that the experiment can be replicated across centers and over time [53]. This desired objectivity is based on the involvement of systematic recourse to the collective production of evidence and requires standardization of practices within settings in order to produce replicable findings which can then be used to standardize practice within, and across, clinical care settings [54]. Moreover, efforts will be made to increase the number of participants in each setting in order to reach a certain degree of comparable statistical weight for every practice. Throughout the implementation phase, information about interventions offered to each patient will be collected with a standardized checklist filled out by the CDPM professionals after each appointment to ensure the fidelity of the intervention [55].

This study includes an evaluation process that measures the integrity and feasibility of the implementation interventions and clinical interventions that will be used. Furthermore, the study will assess how the interventions are executed, will distinguish between components of the interventions, and will identify contextual factors that may influence the content and effectiveness of the implementation intervention. The process evaluation will also examine how well the interventions were adapted to local barriers and facilitators.

#### Data collection

Based on the categories of participants (patients/families, primary care professionals, specialists, decision-makers), four data collection strategies will be used within in-depth multiple case studies with embedded levels of analysis [56]. The proposed strategies are as shown in Table 2:

a. Focus groups (FG) to examine opinions and reactions and to establish a collective understanding of the evaluation aspects proposed [57]. These FG will be conducted by an experienced interviewer using interview guides with open-ended questions developed for each level of analysis, in order to obtain information on the identified dimensions;

b. Semi-directed individual interviews (II) conducted with specialist physicians and CDPM professionals;

c. Patient files (5 per clinic) will be reviewed, using an extraction grid. This review will serve both to clarify how the range of services at the level of patients has been operationalized, and to highlight elements of services integration;

d. Analysis of documentation: Several documents produced during the intervention implementation (checklist for the intervention fidelity report, summaries of team meetings, etc.) will be analyzed to provide an in-depth understanding of the various contexts in which the intervention was deployed, in order to elucidate the positive elements and barriers that were encountered: committee meeting reports, the clinical project coordinator’s notes, internal and external announcements to various professional and non professional communities, etc.

##### Data analysis

The data collected from all participants (patients/families, primary care professionals, specialists, decision-makers) during individual interviews and focus groups (transcription of audio recordings and observer notes) will be analyzed using content analysis. Following an inductive approach combined with thematic analysis, these analyses will be done in three steps to identify emerging themes and trends. The first step is the “coding” which consists of reading and analyzing the corpus. Data management software (NVivo 9.0, QSR Int. USA) will be used to identify units of meaning that will be grouped into nodes that will help extract information related to the same subject. The second step is “sorting” the information into codes or code combinations that will be reviewed and sorted according to different contexts. This information can then be broken and grouped differently from the original version. The last step is “analysis” that will be done throughout and after the coding process. This phase will allow us to analyze all the coded extracts from various documents. In addition to revealing the specific elements of each of the implementation assessment dimensions that were chosen, analysis at this stage will specifically seek to deepen our understanding of the interaction between the intervention, its context and its effects. Contextual determinants of the changes will be investigated using various explanatory models, including political, structural and psychological ones [48,58].

#### Evaluation of effects (objective 2)

Effects will be assessed using three different strategies. To measure short-term effects, a pragmatic randomized experimental design with delayed intervention in the control group will be used [59]. The first step of randomization is the sequence generation using a simple random allocation sequence. The second step is allocation concealment by development and allocating concealment mechanism (sequentially numbered, sealed, opaque envelopes) followed by preparation of the allocation concealment mechanism using the sequence from step one. Research team members involved in generation and allocation concealment will be different from the team members involved in the implementation of assignments. Thus, the intervention group allocation system is set up so that the team member enrolling participants does not know in advance in which group the next person will be allocated.

Patients will be referred by primary care providers (family physician or nurse) to the research team who will be responsible for assessing eligibility and obtaining informed consent, as well as baseline measures (T1). For each study participant, a research assistant at the central office will open a numbered and sealed envelope. The card inside will tell if the patient will be in group A or in group B. This information will immediately be provided to the patient and subsequently to the clinical team in order to schedule the clinical interventions.

Because participants will be invited to receive the interventions in their usual clinics, blind randomization is not possible. Both patients and health care providers will know who is involved in each group, therefore we used independent (unmatched) random samples of patients to receive the intervention immediately after baseline assessments (Group A: immediate intervention group) or at the end of a three-month waiting period (Group B: delayed intervention group). We chose this randomization process because of the potential proximity of patients in and between clinics. In practice, however, we think that communication between patients of the same clinic or between clinics is scarce and will not pose a substantial threat for contamination.

Since it is possible that participants in different groups and phases might come in contact with each other as many will be recruited in the same practice, some precautions will be taken in order to minimize the before and after evaluation biases. To prevent selection bias, the rigorous randomization methodology and allocation concealment process described previously will be respected throughout the project. The methods were reviewed and approved by the Research Ethics Board of the CSSSC and the measurement tools were validated. We expect a response rate greater than 80% and baseline measurements will be comparable in the sense that no significant differences will exist between the groups with respect to age, gender and pre-test measurements. If necessary, the statistical models will be adjusted to ensure comparability of the groups. Each subject intervention will last less than three months and the clinical intervention period is limited to 12 months in order to ensure a contemporaneous data collection.

As stated in the logic model, with the three-month delay design we seek to examine: (1) whether the immediate intervention group (Group A) will have changes at 3 months that are sustained; (2) whether there will be changes in the first 3 months before the intervention with Group B; (3) whether the short-term results of Group A will be different from those of Group B; and (4) whether the outcomes of Group A can be sustained one year later. Theoretically, it is anticipated that these short-term results in attitudes and behaviors of patients will be the precursors of the mid-term changes expected in the maintenance of a healthy lifestyle, quality of life and psychological well-being.

In each clinic where the intervention will be deployed, patients eligible to receive the intervention must be between 18 and 75 years of age and present at least one of the following conditions: diabetes, cardiovascular disease, chronic obstructive pulmonary disease (COPD), asthma or risk factors (smoking, obesity, dyslipidemia, glucose intolerance, and metabolic syndrome). Patients with serious cognitive problems will be excluded.

The decision to explore all these chronic conditions together was preferred to the single disease design for many reasons. The first reason is that this project is an adaptation of CD specialized services already deployed in the region for these chronic conditions into an integrated network. Although these services have often been cited as an example of the successful integration of CDPM services, this approach still faces several challenges requiring a review of its operations and organization to better reflect the reality of patient follow-up and needs. To ensure greater accessibility and better post-intervention continuity, it has been noted that closer collaboration with primary care professionals such as family physicians and nurses from various primary care settings will be necessary in the next few years. Thus, all services for patients with a CD could be provided in an integrated and ongoing fashion. The second reason is that multifaceted interventions are more effective than single ones [60], and sustainable change requires a multi-level approach especially when multiple interventions at varying levels are interlinked and mutually reinforced to maximize the impact on patient behaviour, life-style and self-management. This approach involves action at the patient, practitioner, health care provider and service organization levels in delivery and support. This study aims to provide guidance to patients through trained practitioners working within a health care system that must be responsive to long-term patient needs through its services and patient-centered approach. Accordingly, we have adapted the CDPM professional training so the interventions can be delivered in primary care settings. The selection of these targeted CD (diabetes, cardiovascular disease, asthma, COPD and risk factors for these diseases) is based on a pragmatic approach that aims to enrol patients whose characteristics are similar to those most frequently seen in primary health care settings.

To comply with the pragmatic nature of the intervention, it is up to the referring professional (primary care physician or nurse) to determine eligibility. For each patient, the referring professional will request the patient’s authorization to disclose his or her contact information to the research team and then complete a referral form. Patients refusing to take part in the study will still be eligible to receive the intervention without being involved in the evaluation process. Two patients from the same family can receive the intervention, but only one (randomly selected) will be asked to participate in the research project.

Patients agreeing to participate will complete an initial set of questionnaires at baseline (T1) collecting the main variables and sociodemographic data. Patients will be randomized to receive either the intervention within a short period of time (Group A) or later (three months later for Group B). At time 2 (T2), which will occur three months after the intervention for Group A or immediately before the intervention for Group B, patients will complete the second evaluation questionnaire. Baseline measures will be used to document equivalence between groups while T2 measures will enable the assessment of the effect after three months. A short questionnaire documenting co-interventions will also be administered at T2 (Table 3). Co-interventions represent any additional health care or CDPM services and/or therapeutic procedures other than those that are formally included in the study and may introduce confounding variables that could potentially affect the validity of the results of this study.

**Table 3:** Variables and outcome measures

**Short-term:** Key measurements of effect (measured at T1 and T2 in Groups A and B)	
Self efficacy	The Self-Efficacy for Managing Chronic Disease (SEM-CD) scale (6 items) developed by the Stanford Patient Education Research Center [67].
Empowerment	Proxy of “The Health Education Impact questionnaire (heiQ)” [77]
Comorbidity	The Disease Burden Morbidity assessment (DBMA) [64]
**Mid-term:** Key measurements of effect (measured at T1 and T3 in Groups A and C)	
Health behaviour	Behaviour Risk Factor Survey System Questionnaire (eating habits, physical activity, smoking and alcohol consumption) [65]
Functional health status/quality of life	SF -12 [63,78]
Psychological well-being	K-6 [62]
Comorbidity	The Disease Burden Morbidity Assessment (DBMA) [64]
**Other variables**	
Participant characteristics	Sex, age, socioeconomic status, education, referral diagnosis
Co-intervention	Questionnaire on co-interventions

**Figure 2:**
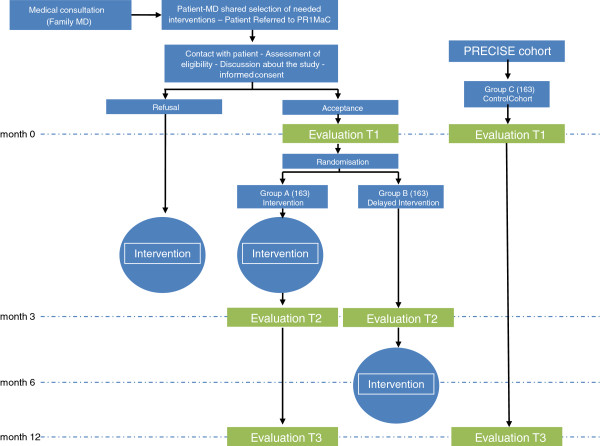
Study timeline.

To document the effect over a year (mid-term), a repeated measures design is proposed. Thus, all patients in Group A will be reassessed a third time (T3), one year after T1. The T3 questionnaire will be identical to the one used at T1 and T2 (Figure 2).

Finally, a quasi-experimental design with a non-equivalent group will be used to evaluate mid-term effects. The control group (Group C) will come from the PRECISE research program platform (main investigators: Jeannie Haggerty and Martin Fortin, http://www.programmeprecise.ca) [61]. The PRECISE study is a cohort of 2197 adults aged 25-to-75 years followed for 4 years. The target population is community-dwelling adults undifferentiated by disease, who sought primary health care locally, did not suffer from major cognitive impairment, and were able to respond to written and oral questions in English or French. Participants were randomly selected within the geographic boundaries of four local health care networks in Québec (Canada). At recruitment (T0), cohort participants reported on sociodemographic information, functional health and health care use. Two weeks (T1), 3 months (T2) and 12 months (T3) after recruitment, they completed a self-report questionnaire on their current health, health behaviours and primary health care experience in the previous year. Use of medical services is confirmed through the review of administrative databases. As in the present study, the PRECISE cohort’s main dependent variable is functional health status and quality of life which are measured with the second version of the Short-Form-12 survey (SF-12v2) [62,63]. Comorbidity is measured using the validated Disease Burden Morbidity Assessment [64]. Lifestyle and health behaviour (eating habits, physical activity, smoking and alcohol consumption) are measured using the Behavioral Risk Factor Survey System Questionnaire [65]. This unique longitudinal cohort in Canada that is providing valuable information on the effectiveness of care in the general population rather than in clients of selected care models will offer us the opportunity to measure health care experience and to quantify the impact of introducing different models of CDPM services.

Patients from this cohort will be matched with patients from Group A by age, gender, diagnosis and family income. Groups will be compared on the basis of changes over a year.

##### Variables and outcome measures

The variables presented in this section were selected to estimate the effects based on the study objectives and the logic model of the intervention. The variables that will be collected according to the measurement schedule are enumerated in Table 3.

Sociodemographic characteristics include gender, age, education, revenue, marital status, and occupation. Health status includes physical functioning, role limitations because of physical health problems, bodily pain, general health perceptions, vitality (energy/fatigue), social functioning, role limitations because of emotional problems, and general mental health (psychological distress and psychological well-being). These dimensions will be investigated using the SF-12 that is a multipurpose short-form (SF) generic measure of health status. The SF-12 Health Survey includes 12 questions from the SF-36 [66].

Level of perceived disease-management self-efficacy will be evaluated using the 6-item Self-Efficacy for Managing Chronic Disease (SEM-CD) (Stanford Patient Education Research Centre 2007) [67]. Each item consists of a question asking how confident the subject feels in different aspects of disease self-management. The observed range is from 1 to 10, with a higher number indicating a greater level of perceived disease-management self-efficacy. The score for each item is summed and then averaged to yield the mean self-efficacy score. This self-efficacy score has been shown to be responsive to change following intervention in chronic disease self-management [68].

The Health Education Impact Questionnaire (HeiQ) provides a broad profile of the potential impacts of patient education interventions [69]. HeiQ evaluates health education (imparting skills) impact as well as larger psychosocial (empowerment) impact.

The Kessler psychological distress scale K-6 is a measure of nonspecific psychological distress that is sensitive to discriminating community DSM-IV cases from non-cases in the general population [70,71]. K-6 is a score derived from the sum of the scores of the responses to each of the six questions on mental illness.

The Disease Burden Morbidity Assessment (DBMA) and a questionnaire on health behaviours (eating habits, physical activity, smoking and alcohol consumption) will be used to characterize patients in different groups. Moreover, referral diagnoses, comorbidities and selected interventions will be evaluated in each group.

##### Data analysis

We will first describe participants’ characteristics in each group, using means and standard deviations for continuous variables, and percentages for categorical variables. The *t*-test (for continuous variables) and the chi-square test (for categorical variables) will be used to compare baseline characteristics across study groups.

To evaluate short-term effects, Groups A and B will be compared on T2 scores (continuous variables such as self-efficacy, health education impact) with an analysis of covariance (ANCOVA) adjusted for T1 scores [72]. If the groups differ at baseline despite randomization, the ANCOVA will also be adjusted for the relevant variables [73]. The independent variable “Group” will be tested for significance at the 5% level.

To document effects over a year, a repeated measures analysis of variance will be used to study the evolution of continuous variables collected three times in Group A [74]. Mid-term effects will be evaluated by comparing T3 measurements in Group A (modifiable risk factors, functional health status and psychological well-being) with the same variables measured in Group C (from the PRECISE cohort) using an ANCOVA adjusted for T1 scores and non-equivalent baseline characteristics (variables not used for matching) [73]. Since groups A and C will be matched on four variables only, they will be considered independently in the analysis. Again, the variable “Group” will be tested for significance at the 5% level.

##### Sample size and statistical power

The required sample size for the randomized clinical trial was calculated for the two main variables (measured with the SEM-CD and the heiQ) with a two-sided α = 0.05 and 80% power. First, for continuous scores, 64 participants in each group will allow to detect a medium effect size (ES=0.5) [75]. In addition, for the heiQ, results are also expressed as the percentage of patients improving at least half a standard deviation. In a before–after study, 35% of patients receiving the intervention presented an improvement of this magnitude [69]. Using these data in a conservative scenario providing for improvement in 20% of control participants, 138 patients are required in each group to detect a 15% difference. Accounting for an anticipated drop-out rate of 15%, 326 patients will be randomized, 163 in each group. A smaller ES of 0.34 will thus be detectable for continuous scores.

Comparison of the 138 subjects in Group A, re-measured at T3 to the same number of subjects selected in the PRECISE cohort, will also allow detection of an ES of 0.34 when measuring mid-term effects (α = 0.05, power = 80%). An additional 15% will also be enrolled in anticipation of withdrawals.

### Limitations and biases

Although conducting a randomized clinical trial may present a challenge in clinical settings where some patients receive an intervention (Group A) while others are waiting for the intervention (Group B), the short period of time between randomization and the second measurement will limit the possibility of co-interventions and maturation biases. Nonetheless, given the pragmatic nature of the trial, patients will be free to seek information beyond the proposed intervention. This may contribute to improve the observed effect. Co-interventions will be evaluated by questionnaire and will be taken into account in the analysis. In addition, analysis of the implementation will bring qualitative insight to this phenomenon. The repetition of the questionnaires may induce a learning effect; however, the time between each execution is long enough to reduce it.

The study design and its short term outcomes measure have been developed specifically to address the problem of the short period between T1 and T2. The project’s proposed two-year timeframe does not allow any exploration of the long-term efficacy of interventions on the use of the health care system.

The intervention may differ from one setting to another, but action will be taken to minimize this risk. We will outline the major considerations that may contribute to a variation in intervention or its fidelity at each stage.

The decision to measure psychosocial outcomes only is based on theoretical reasons that such outcomes are particularly pertinent to examine the adoption and maintenance of a healthy lifestyle and a better quality of life.

One potential bias is the recruitment bias, where health care providers recruit differently depending on their practice, which leads to selection bias and lack of comparability [76]. Recruitment for the present study is prior to allocation which will be provided by the research team. We will pay particular attention to find out whether patients are comparable from one participating setting to another as each primary health care professional will select and refer his or her patients and each participating setting has its own distinct contextual variables.

### Feasibility

After a six-month implementation phase, the study will take place over 18 months and will involve eight medical clinics. To recruit 326 patients for the study and meet the minimal time between intervention and effects in one year, patient selection will need to be done within six months. At the very conservative mean rate of two patients per week per clinic, this objective should be easily achieved. Pairing 138 patients with the PRECISE cohort with a potential pool of 2197 patients from the waiting rooms of medical practices is feasible. With regard to health care providers in clinical settings, a survey was conducted in September 2010 that showed their willingness to participate.

### Ethical considerations

The project received approval by the research ethics committee of the Centre de santé et de services sociaux de Chicoutimi. Informed consent will be obtained from all participants. For ethical reasons, patients who decline to participate will still be offered the intervention in their clinic. For patients willing to participate, the three-month waiting period for the delayed intervention group (group B) will have a negligible effect, given the chronic nature of their conditions. Informed consent will be sought from each participant for the use of administrative data. Confidentiality will be ensured and the data will be stored according to the rules and recommendations of the research ethics committee.

## Discussion

In the short-term, we are expecting improved patient self-efficacy, empowerment and self-management. In the long-term, this should result in a reduction of their risk factors, with an improvement in quality of life and psychological distress. At the organization level, the project should lead to coordinated service delivery, improved patient follow-up mechanisms and enhanced interprofessional collaboration.

Health care program evaluation represents an important step in the adaptation of evidence-based medicine to primary health care reality. Evaluations should be planned, conducted, and reported in ways that facilitate follow-through by stakeholders and decision-makers. Reports should clearly describe the health care program being evaluated, including its context, purposes, procedures, findings and recommendations, so that essential information is provided and easily understood. Patients with CD are among the highest users of health care services. It is important to increase our understanding of primary health care needs for CDPM services and the characteristics of conceptual models of interventional approaches designed for patients with CD who are followed by family physicians.

The study protocol aims to adapt and integrate CDPM services into primary health care settings and to use innovative strategies to evaluate the processes and effects of such interventions.

In conclusion, the integration of CDPM services in primary health care practices is a promising care delivery innovation that needs to be thoroughly evaluated. Successful implementation of health care reform requires new concepts and directions that are strongly supported by objective outcome measures. Continuous program evaluations will facilitate the achievement of the primary goal of the health care improvement debate: high-quality care for every patient notwithstanding the nature or number of his or her diseases.

## Abbreviations

ANCOVA: Analysis of covariance; ANOVA: Analysis of variance; CCM: Chronic care model; CD: Chronic diseases; CDPM: Chronic diseases prevention and management; COPD: Chronic obstructive pulmonary disease; CVD: Cardiovascular disease; FG: Focus groups; II: Individual interviews; PRECISE: Program of research on the evolution of a cohort investigating health system effects.

## Competing interests

Dr Martin Fortin holds Canada’s CIHR Applied Research Chair on Health Services and Policy Research on Chronic Diseases in Primary Care. This project is funded by the Fonds Pfizer-FRSQ-MSSS sur les maladies chroniques. None of the funding agencies had any role in preparing, reviewing or approving the manuscript. They will not be involved in the collection, analysis or interpretation of the data.

## Authors’ contributions

MF and MCC contributed to the conception and design of the study, performed major parts of the review and wrote the draft manuscript. TB participated in the revision of this manuscript. MFD wrote the statistical methods and reviewed drafts of the manuscript. CG and MB reviewed and commented drafts of the manuscript. All authors read and approved the final manuscript.

## Pre-publication history

The pre-publication history for this paper can be accessed here:

http://www.biomedcentral.com/1472-6963/13/132/prepub
